# Paediatric haemolytic uraemic syndrome related to Shiga toxin-producing *Escherichia coli*, an overview of 10 years of surveillance in France, 2007 to 2016

**DOI:** 10.2807/1560-7917.ES.2019.24.8.1800068

**Published:** 2019-02-21

**Authors:** Mathias Bruyand, Patricia Mariani-Kurkdjian, Simon Le Hello, Lisa-A King, Dieter Van Cauteren, Sophie Lefevre, Malika Gouali, Nathalie Jourdan-da Silva, Alexandra Mailles, Marie-Pierre Donguy, Estelle Loukiadis, Delphine Sergentet-Thevenot, Chantal Loirat, Stéphane Bonacorsi, François-Xavier Weill, Henriette De Valk, Réseau français hospitalier de surveillance du SHU pédiatrique

**Affiliations:** 1 Santé publique France, Saint Maurice, France; 2 Service de Microbiologie, Hôpital Robert-Debré, AP-HP, Paris, France; 3 Institut Pasteur, Unité des Bactéries Pathogènes Entériques, Centre National de Référence des *E. coli*, *Shigella* et *Salmonella*, Paris, France; 4 Santé publique France, Rennes, France; 5 Ministry of Agriculture, Agrifood, and Forestry, Paris, France; 6 Université de Lyon, VetAgro Sup, Laboratoire National de Référence pour les Escherichia coli, Marcy l’Etoile, France; 7 Université de Lyon, CNRS, INRA, Université Claude Bernard Lyon 1, VetAgro Sup, Laboratoire d’Ecologie Microbienne, Villeurbanne, France; 8 Pediatric Nephrology Department, University Hospital Robert Debré, Paris, France; 9 Members of the Réseau français hospitalier de surveillance du SHU pédiatrique have been listed at the end of the article

**Keywords:** haemolytic uraemic syndrome, HUS, children, surveillance, Shiga toxin producing Escherichia coli, E coli, France, food-borne infections

## Abstract

**Introduction:**

Haemolytic uraemic syndrome (HUS) related to Shiga toxin-producing *Escherichia coli* (STEC) is the leading cause of acute renal failure in young children. In France, HUS surveillance in children aged < 15 years was implemented starting from 1996.

**Aim:**

We present the results of this surveillance between 2007 and 2016.

**Methods:**

A voluntary nationwide network of 32 paediatric departments notifies cases. Two national reference centres perform microbiological STEC confirmation.

**Results:**

Over the study period, the paediatric HUS incidence rate (IR) was 1.0 per 100,000 children-years, with a median of 116 cases/year. In 2011, IR peaked at 1.3 per 100,000 children-years, and decreased to 1.0 per 100,000 children-years in 2016. STEC O157 associated HUS peaked at 37 cases in 2011 and decreased to seven cases in 2016. Cases of STEC O26-associated HUS have increased since 2010 and STEC O80 associated HUS has emerged since 2012, with 28 and 18 cases respectively reported in 2016. Four STEC-HUS food-borne outbreaks were detected (three STEC O157 linked to ground beef and raw-milk cheese and one STEC O104 linked to fenugreek sprouts). In addition, two outbreaks related to person-to-person transmission occurred in distinct kindergartens (STEC O111 and O26).

**Conclusions:**

No major changes in HUS IRs were observed over the study period of 10 years. However, changes in the STEC serogroups over time and the outbreaks detected argue for continuing epidemiological and microbiological surveillance.

## Introduction

Shiga toxin-producing *Escherichia coli* (STEC) causes bloody diarrhoea, which is complicated in 5–15% of paediatric patients by haemolytic uraemic syndrome (HUS) [1]. HUS is defined by the triad of mechanical haemolytic anaemia, thrombocytopenia and acute kidney injury [1]. STEC related HUS, also called post-diarrhoeal HUS, is the main cause of acute renal failure in young children [1]. The thrombotic microangiopathy process mainly affects the kidney, potentially resulting in long-term sequelae. Other organs such as the intestine, central nervous system and heart may also be affected [2]. Ruminants, especially cattle, are the main reservoirs for the highly virulent STEC O157 strain [3], as well as other HUS-associated non-O157 strains [4]. STEC infection routes are ingestion of contaminated food or water, contact with an animal or an environment contaminated by STEC (such as bathing water or soil in the proximity of ruminants) [5,6] or person-to-person transmission, which is frequently observed due to the low infectious dose of STEC (< 1,000 organisms) [7]. Due to the outbreak potential and the severe morbid conditions related to STEC HUS, STEC infection represents an important public health concern [1,8,9]. Various options are available for surveillance, which can be based on case notification on a mandatory or voluntary basis, and microbiological surveillance of STEC infection relying on a National Reference Centre (NRC) for human samples [10-12]. In France, STEC surveillance relies on voluntary HUS surveillance in children aged < 15 years [13-15]. STEC identification in stool samples is not routinely performed by medical laboratories in France, however any laboratory in the country can send a stool sample to the associated laboratory of the NRC for STEC screening, highlighting the key role of the NRC for human samples in STEC surveillance.

This report presents the results of the French paediatric HUS surveillance over a 10-year period, from 1 January 2007 to 31 December 2016.

## Methods

### Description of the French paediatric haemolytic uraemic syndrome surveillance system

The French paediatric HUS surveillance system is implemented since 1996. It is coordinated by Santé publique France, the French national public health agency.

The objectives of the surveillance system are to monitor the spatial and temporal trends of paediatric HUS incidence, describe the characteristics of cases, and detect clusters of cases to guide prevention and control measures.

Children aged less than 15 years presenting with the triad of acute renal failure, mechanical haemolytic anaemia, and thrombocytopenia fulfil the case definition criteria and are notified to Santé publique France. Acute renal failure is defined as serum creatinine levels above 60 µmol/L in children aged less than 2 years and above 70 µmol/L in older children. Mechanical haemolytic anaemia is defined as haemoglobin level lower than 10 g/100 mL, with a proportion of schistocytes ≥ 2%. Thrombocytopenia is defined by a platelet count lower than 150,000/µL.

Thirty-two French paediatric departments distributed throughout France (excluding the overseas departments), including all the 21 university hospital units specialised in paediatric nephrology, are involved in the surveillance since 1996. Physicians notify HUS cases in real time on a voluntary basis to Santé publique France. In addition, any physician or microbiologist in France, may notify cases of HUS or STEC infection to Santé publique France, regardless of their routine involvement in the surveillance system. Notified cases reporting travel abroad during the 7 days preceding onset of symptoms are considered as imported and not included in the surveillance reports.

### Confirmation of Shiga toxin-producing *Escherichia coli* infection or environmental contamination

The NRC for *Escherichia coli, Shigella* and *Salmonella* in human samples (Institut Pasteur, Paris, France) and its associated laboratory (Microbiology laboratory, University Hospital Robert Debré, Paris, France) confirm STEC infection. Confirmation is done through the detection by PCR in stool samples of the virulence genes for the Shiga toxins (*stx1* and *stx2* genes), the gene for the outer membrane protein intimin (*eae* for *E. coli* attaching and effacing), the enterohemolysin gene (*ehxA*), and the O-antigen biosynthesis genes of the 10 most frequent STEC serogroups affecting humans in France (O157, O26, O145, O55, O103, O104, O111, O91, O121, and O80) [15]. STEC screening in stool is considered positive if *stx1* and/or *stx2* genes are detected. Culture is performed on all *stx* positive stools to characterise STEC strains by serogrouping and to test for the same virulence genes. Non-typeable STEC strains are characterised by using PCR-restriction fragment length polymorphism of the O operon (rfb-RFLP) and sequencing of the flagellin gene (*fliC)* [16,17]. Investigations for the presence of STEC strains in food and environmental samples are performed by the National Reference Laboratory (NRL) for *E. coli* isolated from food and environmental sources (VetAgro Sup, Lyon, France) according to the XP CEN ISO/TS 13136:2012 method [18]. Serotyping of the STEC strains isolated is performed by PCR as described previously [16,17].

### Information routinely collected

We systematically collect data on the date of symptom onset, gastrointestinal symptoms, biological parameters related to HUS and STEC strain, and outcome using a standardised notification form. Information about related cases of HUS or diarrhoea, and exposure to traditional STEC risk factors in the 2 weeks preceding HUS diagnosis (raw milk dairy or ground meat consumption, contact with farm animals, bathing in a pool or surface water) are collected as well. Epidemiological investigations are not routinely performed to identify the infection source of sporadic cases. Thus, information routinely gathered on the notification form allows describing cases’ characteristics rather than identifying cases’ infection sources.

### Investigations of suspected clusters

Any suspicion of a cluster of HUS or diarrhoea cases potentially due to STEC infection leads to an epidemiological investigation to confirm or rule out an outbreak, identify the source of infection and guide control measures. We assess potential STEC exposures within the 7 days preceding symptom onset using a detailed trawling questionnaire completed with the parents or caretakers of the child. For the specific cases related to the STEC O104:H4 strain, the investigation covers the 14 days preceding symptom onset, as the incubation period for this specific serogroup may exceed 1 week [19,20].

An outbreak is defined as two or more HUS or STEC infection cases with an epidemiological link (common exposure or person-to-person transmission). In an outbreak context, the NRC for human samples and the NRL for *E. coli* isolated from food and environmental sources are involved through confirming STEC infection in cases, identifying STEC in the suspected sources, and comparing STEC strains isolated in cases’ stools and in suspected sources of infection. The strain comparisons are performed using the Standard PulseNet PFGE protocol for *E. coli* O157 [21], and increasingly, since 2016, using whole genome sequencing (WGS) methods.

### Population estimates

Population estimates provided by the National Institute for Statistics and Economic Studies were used to assess incidence rates of reported HUS (IR) [22].

## Results

**Figure 1:**
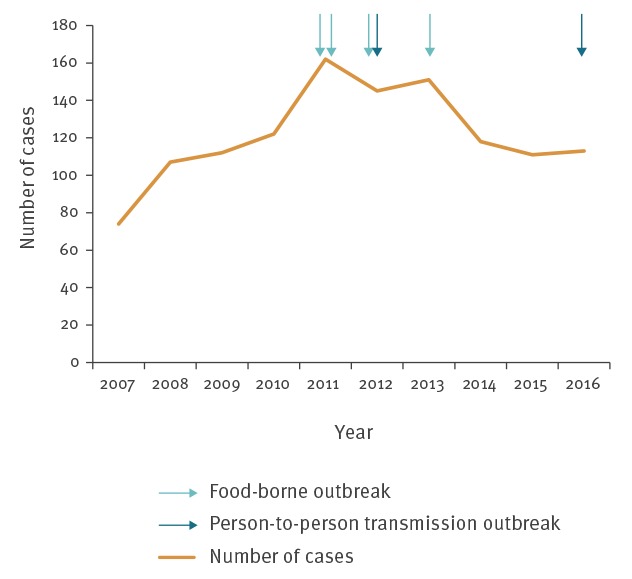
Numbers of paediatric haemolytic uraemic syndrome cases reported to the surveillance system and numbers of outbreaks detected, France, 2007–2016 (n = 1,215 cases)

From 2007 to 2016, a median of 116 paediatric HUS cases was reported each year (minimum: 74; maximum: 162) (Figure 1). The highest annual IR was reported in 2011 with an IR of 1.3 cases per 100,000 children-years, in 2016 the IR was 1.0 case per 100,000 children-years. During the study period, a total of 1,215 cases were notified (IR of 1.0 case per 100,000 children-years), including 32 (3%) who were part of six recognised outbreaks. Of these outbreaks, four were food-borne, including two in 2011, one in 2012 and one in 2013. A temporary increase of the reported number of sporadic cases (not linked to the outbreak) was observed during the months following each of these outbreaks. The remaining two outbreaks involved person-to-person transmission and occurred in two different kindergartens in 2012 and 2016 (Figure 1).

HUS: haemolytic uraemic syndrome.

The numbers of children with HUS were one and 18 during the two outbreaks in 2011, respectively; four and four during the two outbreaks in 2012; four during the outbreak in 2013 and one during the one in 2016.

**Figure 2:**
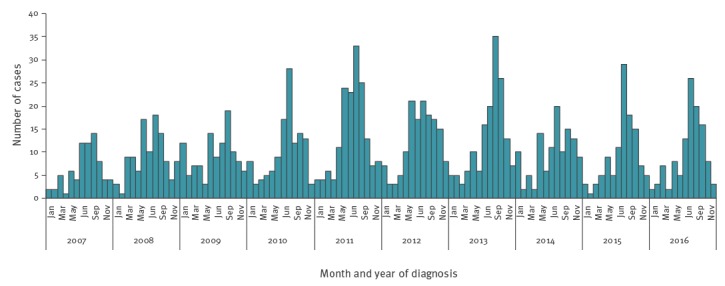
Monthly number of reported paediatric haemolytic uraemic syndrome cases, France, 2007–2016 (n = 1,215 cases)

Seasonality was consistently observed, with the majority of cases being reported in the summer months (Figure 2). In this period, the number of notifications was usually two to three times higher compared with the winter months.

**Figure 3:**
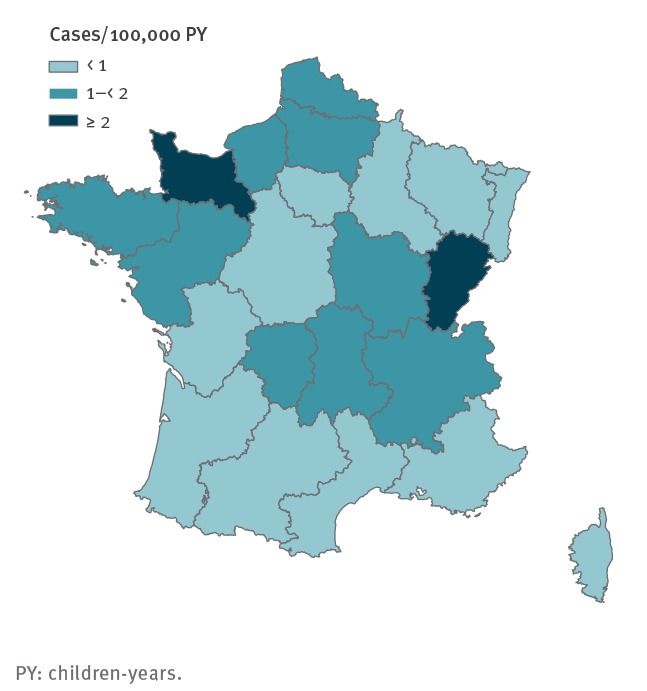
Incidence of paediatric haemolytic uraemic syndrome by region, France, 2007–2016

Paediatric HUS IR demonstrated an important spatial heterogeneity. During the study period, the most affected regions were located in north-west and central-eastern France (Basse-Normandie and Franche-Comté), with an IR more than double the least affected regions (south, centre and north-east of France) (Figure 3).

PY: children-years.

In children aged less than 6 months, IR was 0.8 case per 100,000 children-years. IR peaked to 3.3 cases per 100,000 children-years in children aged 6 months to 2 years (35 months) and then decreased with increasing age: 1.2 cases per 100,000 children-years in children aged 3 to 5 years, 0.5 case per 100,000 children-years in those aged 6 to 10 years and 0.2 case per 100,000 children-years in those aged 11 to 14 years. There was no difference in IR according to sex (IR of 1.0 case per 100,000 children-years in boys and 1.1 cases per 100,000 children-years in girls).

Median age at HUS diagnosis was 30 months (interquartile range (IQR): 17–62). Over the entire study period, HUS was preceded by diarrhoea in 1,099 (90%) cases, of whom 545 (50%) had bloody diarrhoea (Table 1). The median delay between diarrhoea onset and HUS diagnosis was 6 days (IQR: 4–8). During the entire study period, acute renal failure was observed in 71% of cases, median plasma creatinine level was 188 µmol/L (IQR: 73–389) and median blood platelet count was 39,000/µL (IQR: 23,000–65,000). Half (49%, 592/1,215) of patients required blood transfusion, and 32% (389/1,215) required dialysis. Eleven deaths (0.9% of cases) were reported. No major changes were observed in the characteristics of cases over the study period (Table 1). Renal failure seemed to be more frequent in 2007–08 (86% vs 64% to 71% between 2009 and 2016). Over the study period, the highest values of creatininaemia (median: 217 µmol/L; IQR: 103–440) were also observed in 2007–08 (Table 1). In 2007–08, 38% of children had dialysis, and this proportion ranged between 29% and 33% between 2009 and 2016 (Table 1).

**Table 1:** Characteristics of paediatric HUS cases reported to the French HUS surveillance system, France, 2007–2016 (n = 1,215 cases)

Characteristic	2007–2008			2009–2010			2011–2012			2013–2014			2015–2016			Total		
	Median (IQR)	n	%	Median (IQR)	n	%	Median (IQR)	n	%	Median (IQR)	n	%	Median (IQR)	n	%	Median (IQR)	n	%
HUS	NA	180	100	NA	234	100	NA	307	100	NA	269	100	NA	225	100	NA	1,215	100
Male sex	NA	97	54	NA	112	48	NA	164	53	NA	121	45	NA	99	44	NA	593	49
Age (months)	33 (19–61)	NA	NA	31(18–60)	NA	NA	35 (18–71)	NA	NA	29 (15–59)	NA	NA	24 (14–54)	NA	NA	30 (17–62)	NA	NA
Diarrhoea	NA	171	95	NA	220	94	NA	277	90	NA	242	90	NA	189	84	NA	1,099	90
Bloody diarrhoea	NA	99	55	NA	112	48	NA	155	50	NA	92	34	NA	87	39	NA	545	45
Delay diarrhoea to HUS (days)	5 (4–7)	NA	NA	6 (4–9)	NA	NA	6 (4–9)	NA	NA	6 (4–9)	NA	NA	6 (4–9)	NA	NA	6 (4–8)	NA	NA
Acute renal failure^a^	NA	155	86	NA	165	71	NA	210	68	NA	171	64	NA	157	70	NA	858	71
Creatininaemia (µmol/L)	217 (103–440)	NA	NA	211 (78–420)	NA	NA	163 (78–362)	NA	NA	161 (59–397)	NA	NA	181 (61–350)	NA	NA	188 (73–389)	NA	NA
Platelet count (g/L)	39 (23–65)	NA	NA	38 (24–66)	NA	NA	39 (23–65)	NA	NA	40 (26–63)	NA	NA	38 (20–63)	NA	NA	39 (23–65)	NA	NA
Dialysis	NA	69	38	NA	67	29	NA	100	33	NA	78	29	NA	75	33	NA	389	32
Blood transfusion	NA	143	79	NA	84	36	NA	46	15	NA	158	59	NA	161	72	NA	592	49
Death	NA	2	1.1	NA	1	0.4	NA	5	1.6	NA	1	0.4	NA	2	0.9	NA	11	0.9
Stool analysis	NA	123	100	NA	170	100	NA	244	100	NA	225	100	NA	180	100	NA	942	100
*E. coli* O157	NA	38	31	NA	40	24	NA	65	27	NA	50	22	NA	19	11	NA	212	23
*E. coli* O80	NA	0	0	NA	6	4	NA	7	3	NA	23	10	NA	37	21	NA	73	8
*E. coli* O26	NA	9	7	NA	14	8	NA	23	9	NA	21	9	NA	39	22	NA	106	11
*stx1*	NA	9	NA	NA	8	NA	NA	24	NA	NA	27	NA	NA	17	NA	NA	85	NA
*stx2*	NA	64	NA	NA	101	NA	NA	175	NA	NA	179	NA	NA	156	NA	NA	675	NA
*eae*	NA	53	NA	NA	90	NA	NA	163	NA	NA	161	NA	NA	136	NA	NA	603	NA
*ehxA*	NA	NA	NA	NA	37	NA	NA	103	NA	NA	146	NA	NA	123	NA	NA	409	NA

*eae*: *Escherichia coli* attaching and effacing gene; *E. coli*: *Escherichia coli*; *ehxA*: enterohemolysin gene; HUS: haemolytic uraemic syndrome; IQR: interquartile range; NA: not available; *stx*: Shiga toxin gene.

^a^ Acute renal failure is defined as serum creatinine levels above 60 µmol/L in children aged less than 2 years and above 70 µmol/L in older children.

Stool analysis results were available for 942 cases, 730 (77%) of them had a positive STEC result screening. Among 689 cases with a positive STEC screening for whom detailed information was available, the virulence genes *stx1, stx2, eae* and *ehxA* were carried by 12% (85/689), 98% (675/689), 88% (603/689) and 59% (409/689) of the cases respectively. Both *stx1* and *stx2* were detected in 10% (71/689) instances.

Among 942 cases with a stool analysis result available, STEC serogroup was identified in 496 instances. During the study period, the most frequent STEC serogroups identified were O157 (212 cases, 23% of 942 cases with a stool analysis), O26 (106 cases, 11%) and O80 (73 cases, 8%) (Table 1). The most frequents serogroups following the top three were O111 (18 cases), O145 and O55 (14 cases each).

**Figure 4:**
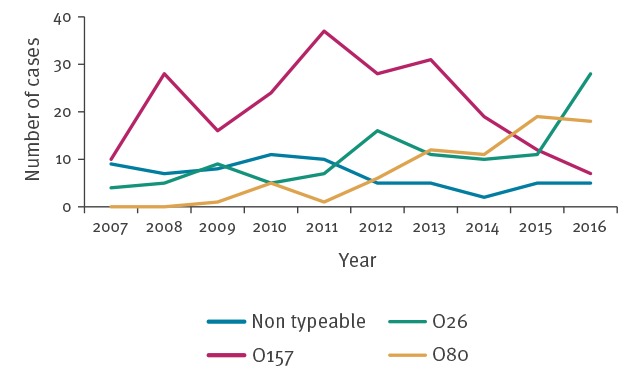
Main Shiga toxin-producing *Escherichia coli* serogroups causing paediatric haemolytic uraemic syndrome identified in stools, France, 2007–2016

Major changes were observed in STEC serogroups over the study period (Figure 4). The number of paediatric HUS cases with a STEC O157 evidenced in stool increased until 2011 (37 cases) and subsequently decreased to seven cases in 2016. From 2014 to 2016, the annual total number of paediatric HUS cases reported remained stable, while the number of cases related to STEC O157 continued to decrease. The O26 serogroup increased after 2010 (5 cases), in 2016 it was the main serogroup evidenced in stool (28 cases). The STEC O80 strain clearly emerged after 2012 to become the main serogroup evidenced in HUS cases’ stool in 2015 (19 cases). The number of cases affected by STEC O80 remained stable in 2016 (18 cases).

Four food-borne outbreaks were detected, involving a total of 56 reported cases including 34 cases of HUS (27 children and 7 adults) [19,23,24]. A STEC O104:H4 outbreak caused by fenugreek detected in 2011 was linked to a large German outbreak, and involved 24 cases in France, of whom six adults and one child had HUS [19,20]. A second outbreak linked to the consumption of contaminated ground beef distributed as frozen burgers also occurred in 2011. Eighteen paediatric HUS cases infected with a sorbitol-fermenting STEC O157:H7 or a STEC O177:H25 were identified by the outbreak investigation [24]. The third outbreak detected in 2012 was related to STEC O157:H7. Six cases including four paediatric HUS cases were linked to the consumption of contaminated fresh ground beef [23]. In 2013, eight cases including four paediatric HUS cases were linked to an outbreak associated with the consumption of raw milk cheese, a non-sorbitol fermenting STEC O157:H- was involved. In the two outbreaks related to contaminated ground beef consumption, enhanced trace-back investigations were performed and led to control measures which consisted of withdrawal and recall of products.

In November 2012, a STEC O111 outbreak occurred in a kindergarten in Brittany. Six confirmed cases were reported, including four paediatric HUS cases, all resulting from person-to-person transmission. Following this episode, the French high council for public health was requested to provide recommendations for the management of STEC gastroenteritis outbreaks in communities such as daycare, schools or retirement homes. These recommendations were published in January 2015 [25]. In 2016, a STEC O26:H11 outbreak occurred in a kindergarten located in the south of France. Seven confirmed cases were reported including one paediatric HUS case. All cases resulted from person-to-person transmission. WGS analysis confirmed that all cases were affected by the same STEC O26:H11 strain.

To explore infection sources of the cases affected by the emerging STEC O80 strain, and assess if this strain’s emergence could be related to an outbreak, epidemiological investigations were implemented for all STEC O80 paediatric HUS cases in 2015. These investigations did not allow identifying common exposures between cases.

Among the total 1,215 paediatric HUS cases notified over the whole study period, HUS and diarrhoea cases were reported among those close to the paediatric HUS case notified in respectively 6% (67/1,215) and 28% (345/1,215) instances, mostly among family members (including adults) in the same household.

Regarding exposure to the main STEC risk factors routinely collected through the notification form, consumption of raw milk was reported by 5% (63/1,215) of cases, raw milk cheese by 22% (263/1,215), ground beef by 54% (655/1,215) (reported as undercooked in 159 instances), bathing in a pool or surface water by 19% (236/1,215), and 20% (247/1,215) reported a contact with farm animals.

## Discussion

Information collected since 1996 has allowed for the description of spatial and temporal trends in paediatric HUS incidence. In addition the description of the characteristics of cases and STEC strains has also been made possible.

We observed strong seasonality and spatial heterogeneity in IR, as previously documented in France between 1996 and 2006 [13]. Seasonality and spatial heterogeneity are also observed in the United States and elsewhere in Europe [10,12].

An important shift occurred in France regarding STEC serogroups evidenced in paediatric HUS cases between 2007 and 2016. STEC O157 has decreased since 2011, while the serogroup O26 has increased since 2010 and the serogroup O80 emerged in 2012. The decreasing number of STEC O157 and the increasing numbers of STEC O26 related HUS cases have also been reported elsewhere in Europe [12]. In 2005, a STEC O157 outbreak related to the consumption of frozen beef burgers involved 69 patients including 17 HUS cases in France [26]. Following this episode, a number of preventive measures in the meat sector were implemented by the French Ministry of Agriculture, Agrifood, and Forestry [26,27]. Especially, STEC management measures in ground meat were enhanced and routine screening for STEC was performed more frequently by food business operators. The decrease of STEC O157-related paediatric HUS cases since 2011 in France could be a consequence of these measures, which also included detailed hygiene regulations for the production of ground meat, as cattle accounts for the main STEC O157 reservoir.

The drivers behind the emergence of the O80 serogroup in France remain unexplained despite enhanced epidemiological investigations performed in 2015 [28]. This emergence raises specific concerns as infection by STEC O80 might lead to previously uncommon STEC related morbidity such as septicaemia [28]. Further studies are needed to better characterise this emerging strain and its determinants. Changes in the STEC serogroups might be associated with changes in the pathogenicity, as observed with the O80 serogroup [28].

In addition to classic STEC food-borne risk factors, previously not documented vehicles such as organic fenugreek seeds (STEC O104:H4) caused outbreaks during the study period [19]. Unexpected vehicles should be taken into account in an outbreak context, especially when traditional risk factors for STEC are not outlined by the initial investigation.

The investigations performed allowed implementing control measures in several instances. Withdrawal and recall of contaminated products where set in the outbreaks linked to contaminated ground beef. Following the first outbreak due to person-to-person transmission occurring in a kindergarten, the French high council for public health provided recommendations to control STEC outbreaks in childcare facilities and schools [25].

The multidisciplinary surveillance network has involved clinicians (mainly paediatric nephrologists), epidemiologists, microbiologists and veterinary public health officers over a 20-year period. Its stability over time is a strength of this surveillance system. It enables clinicians to inform Santé publique France about paediatric HUS cases, and to alert if STEC infection cases are suspected to be clustered.

All the French paediatric units specialised in nephrology are participants of the French paediatric HUS surveillance network since 1996. However, sporadic paediatric HUS cases may be underreported. This is suggested by the temporary increase in the number of sporadic cases notified in the months following outbreaks, probably due to raised awareness among clinicians during these periods. Sensitivity of reporting to the paediatric STEC-HUS surveillance system was estimated to be 66% in 2005 (95% confidence interval: 58–70) [13]. Since 2012 no major STEC HUS outbreak with media coverage occurred in France. This may have resulted in less clinicians’ awareness in the latest years of the study and might have contributed to underreporting. Updated estimations of the sensitivity of the surveillance system would be valuable.

In several countries, STEC-HUS is a mandatory notifiable infection and the United States Centers for Disease Control and Prevention recommends routine stool STEC testing in all diarrhoea cases [29]. In France, most laboratories do not test STEC in stool routinely in patients with diarrhoea unless clinicians specifically request it. The lack of a standard testing panel and protocol for stool cultures and appropriate financial reimbursement of laboratories are also contributing factors to under testing that are difficult to resolve. When performed, microbiological STEC confirmation and strain characterisation relies on the NRC for human samples and its associated laboratory. This allows for the detection of any increase in paediatric STEC-HUS cases related to a given STEC serogroup, as well as clusters scattered throughout mainland France.

The expanding availability of non-culture based methods to identify a panel of infections may increase the number of STEC infections detected. For example, STEC detection can be achieved through multiplex PCR in stool. However, efforts should continue to perform stool culture to isolate STEC strains in all patients with positive *stx* screening in stool. This is the only way we can link scattered cases, especially in a context of expanding availability of WGS. Indeed, WGS enables full STEC strain characterisation and the identification of clusters that cannot be evidenced solely on the basis of epidemiological information, such as geographically scattered outbreaks, or prolonged outbreaks with a persistent source generating a limited increase in the number of cases, but over a long period of time [30]. The importance of early stool collection performed at admission of paediatric HUS cases, or rectal swab when a stool sample is not available, should be highlighted to clinicians.

### Conclusion

The French paediatric HUS surveillance system has found a decline in STEC O157 related paediatric HUS and the emergence of STEC O26 and STEC O80 in France. Six outbreaks were detected and control measures were implemented. This surveillance system is evolving, more widespread stool testing of gastroenteritis cases for STEC and routine use of WGS will improve its ability to further describe STEC strain characteristics and its capacity for outbreak detection.

## Members of the Réseau français hospitalier de surveillance du SHU pédiatrique

Djamal-Dine Djeddi: Paediatric and Adolescent Medicine Departement, Centre Hospitalier Universitaire d'Amiens, Amiens, France.

Lise Allard: Paediatric Departement, Centre Hospitalier Universitaire d'Angers, Angers, France.

Sylvie Roullaud: Paediatric Departement, Centre Hospitalier d’Angoulême, Angoulême, France.

François Nobili: Paediatric Nephrology Departement, Centre Hospitalier Universitaire de Besançon, Besançon, France.

Brigitte Llanas: Paediatric Nephrology Departement, Centre Hospitalier Universitaire de Bordeaux, Bordeaux, France.

Loïc de Parscau: Paediatric Departement, Centre Hospitalier Régional Universitaire Morvan, Brest, France.

Anne-Laure Sellier-Leclerc: Paediatric Nephrology Departement, hôpital Femme-Mère-Enfant, Bron, France.

Philippe Eckart: Paediatric Departement, Centre Hospitalier Universitaire de Caen, Caen, France.

Lucie Bessenay: Paediatric Departement, Centre Hospitalier Universitaire Estaing, Clermont Ferrand, France.

Frédéric Huet: Paediatric Departement, Hôpital d'Enfants, Dijon, France.

Guylhene Bourdat-Michel: Paediatric Departement, Centre Hospitalier Universitaire de Grenoble, Grenoble, France.

Corinne Guitton: Paediatric Departement, AP-HP Centre Hospitalier Universitaire Bicêtre, Le Kremlin-Bicêtre, France.

Robert Novo: Paediatric Nephrology Departement, Hôpital Jeanne de Flandre, Centre Hospitalier Universitaire de Lille, Lille, France.

Vincent Guigonis: Paediatric Departement, Centre Hospitalier Universitaire Limoges, Limoges, France.

Marcel Guillot: Peadiatric Departement, Centre Hospitalier Robert Bisson, Lisieux, France.

Michel Tsimaratos: Paediatric Nephrology Departement, Hôpital de la Timone, Marseille, France.

Marc Fila: Paediatric Departement, Centre Hospitalier Universitaire de Montpellier, Montpellier, France.

Gwenaelle Roussey-Kessler: Paediatric Nephrology Departement, Centre Hospitalier Universitaire de Nantes, Nantes, France.

Etienne Berard: Paediatric Nephrology Departement, Centre Hospitalier Universitaire de Nice, Archet 2, Nice, France.

Tim Ulinski: Paediatric Nephrology Departement, Hôpital Armand Trousseau, APHP, Paris, France.

Chantal Loirat: Paediatric Nephrology Departement, Hôpital Robert Debré, APHP, Paris, France.

Pauline Krug: Nephrology Departement, Hôpital Necker Enfants Malades, APHP, Paris, France.

Violaine Lefranc: Paediatric Emergencies Departement, Centre Hospitalier Universitaire La Miletrie, Poitiers, France.

Catherine Didier-Wright: Paediatric Department, Centre Hospitalier de la Région Annecienne, Pringy, France.

Christine Pietremont: Nephrology Department, American Mémorial Hospital, Reims, France.

Amélie Ryckewaert: Paediatric Nephrology Departement, Centre Hospitalier Universitaire Rennes, Rennes, France.

Françoise Broux: Paediatric Nephrology Departement, Centre Hospitalier Universitaire Charles Nicolle, Rouen, France.

Marie-Pierre Lavocat: Paediatric Departement, Hôpital Nord, Centre Hospitalier Universitaire de Saint Etienne, Saint Etienne, France.

Joelle Terzic: Paediatric Departement, Centre Hospitalier Universitaire de Strasbourg, Strasbourg, France.

Stéphane Decramer: Pediatric Nephrology Department, Centre Hospitalier Universitaire de Toulouse, Toulouse, France.

Hubert Nivet: Pediatric Nephrology Department, Centre Hospitalier Universitaire de Tours, Tours, France.

Isabelle Vrillon: Paediatric Nephrology Departement, Hôpitaux de Brabois, Centre Hospitalier Universitaire de Nancy, Vandoeuvre Les Nancy, France.
